# Self-efficacy reduces the impact of social isolation on medical student’s rural career intent

**DOI:** 10.1186/s12909-018-1142-1

**Published:** 2018-03-20

**Authors:** Vivian Isaac, Sabrina Winona Pit, Craig S. McLachlan

**Affiliations:** 1 Flinders Rural Health South Australia, Flinders University, PO Box 852, Renmark, South Australia 5341 Australia; 2 University Centre for Rural Health, School of Medicine, Western Sydney University, 62 Uralba Street, Lismore, NSW 2480 Australia; 3 School of Public Health, Sydney University, 62 Uralba Street, Lismore, NSW 2480 Australia; 4 Rural Clinical School, Faculty of Medicine, University of New South Wales, Level 3 Samuels Building, Sydney, Australia

**Keywords:** Social cognitions, Rural medical education, Social isolation, Self-efficacy, Medical student, Career intention

## Abstract

**Background:**

Social isolation in medical students is a subjective experience that may influence medical career decision making. Rural self-efficacy has been shown to influence rural career intentions following a rural clinical placement, however its impact on social isolation during a rural clinical placement has not been previously modeled. The objective of this study is to explore whether self-perception of social isolation is associated with rural career intent in rural medical students. Secondly, to determine whether self-efficacy influences the association between social isolation and rural career intent.

**Methods:**

2015 data, from a cross-sectional survey of the National Federation of Rural Australian Medical Educators (FRAME) study. Among 619 medical students attending rural clinical schools (RCS), rural career intent was assessed. This included intended rural location for either postgraduate medical specialist or generalist training or completion of that training. Self-efficacy beliefs in rural medical practice were based on a validated scale consisting of six questions. Social isolation was measured asking students whether they felt socially isolated during their RCS placement.

**Results:**

31.3% of surveyed students self-reported feeling socially isolated during their rural placement. Social isolation was associated with reduced rural career intent after controlling for gender, rural background, RCS preference, RCS support and wellbeing. In step-wise logistic regression the association between social isolation and rural intent disappeared with the inclusion of rural self-efficacy.

**Conclusions:**

Social isolation during a rural clinical placement is commonly reported and is shown to reduce rural career intent. High levels of rural clinical self-efficacy reduce the effects of social isolation on future rural workforce intentions.

## Background

Increasing exposure to a rural clinical environment during medical student training increases Australian rural career interest and intent [1, 2]. This has been observed for both rural and urban entry medical students [1, 3]. Students studying medicine in rural areas are more confident in their clinical skills and show increase interest to practice in a rural setting, possibly through increased access to patients, more hands-on experience and close relationships with patients and colleagues [3–5]. However, a sense of perceived social isolation during a rural placement may reduce the impact of a rural placement on future rural workforce intent.

Perceived social isolation is a subjective experience and may be defined as a sense of not belonging to a community or geographical area. [6]. A sense of social isolation may influence emotions, and behaviors that impact on future motivations and interest in career location. A number of studies suggest that perceived social isolation contributes to increases in depressive thoughts and or distress, [7]. It has been reported that rural medical students whom felt socially isolated during a rural medical school placement were less likely to go on to peruse hospital appointments in rural areas for intern training [8]. Perceived social isolation (as an objective or structural measure) is also associated with health and wellbeing [9]. We note that perceived social isolation as a reflective experience has not been explored in Australian medical students following a rural placement.

A medical student’s level of interest and self-efficacy during a rural clinical placement is associated with future rural medical career intentions [10]. We have previously suggested that vocational interests develop over time, partially as a function of self-efficacy and interest [3]. In rural medical students self-efficacy reflects the beliefs and expectations that one may either feel in the future they can or cannot be a successful medical practitioner in a rural location based on a trial experience. Hence a social cognitive framework can be applied to understand rural career behaviours including self-efficacy [11, 12]. Indeed instruments have been developed to measure self-efficacy in medical students. These instrument measures have been validated and developed to understand medical student career behaviours [10, 13].

A higher level of self-efficacy for rural practice may diminish the effects of perceived social isolation. However, it is unclear, whether in Australian rural medical students, self-efficacy or social isolation, are independent factors with respect to rural career intent. This study aims to determine the incidence of subjective social isolation in medical students during and a rural placement and its effects on rural career intent. We will identify factors that are associated with social isolation in this study population. Secondly, this study aims to explore the effect of rural self-efficacy on the relationship between social isolation and rural career intent.

## Methods

### Design, setting and participants

Data from the 2015 Federation of Rural Australian Medical Educators (FRAME) survey [14] was used for this analysis. These data contained cross-sectional information on 644 medical students who had completed a rural clinical placement across 13 medical universities. Completion of the survey was voluntary and ethics was approved for this study from each of the participating universities.

### Measures

#### Social isolation

Social isolation was measured by students self-reporting whether they felt socially isolated during their rural clinical school placement. “I felt socially isolated during my rural placement”. These responses were assessed on a five-point likert-scale. The answers were dichotomized into: ‘Somewhat agree’ and ‘strongly agree’ versus ‘Strongly disagree’, ‘somewhat disagree’ and ‘neutral’.

#### Rural career intent

Students were asked to identify their preferred location for future practice. “In which geographical location within Australia would you most like to practise on completing your training?” The options were**,** Capital or Major City; Inner regional city or large town in Australia (25,000–100,000); Smaller town - outer regional (10,000–24,999); Small rural or remote communities (10,000) and Very remote centre/area.

#### Rural self-efficacy

Self-efficacy beliefs in rural medical practice were assessed [15]. This tool has been previously described and validated [10]. In summary, six questions that that required responses measured an individual’s self-efficacy to future clinical practice in rural setting. The questions are based on five key career factors of self-efficacy that include vicarious learning, verbal persuasion, positive emotional arousal, and negative emotional arousal and performance accomplishments. From these 6 questions a composite rural medicine self-efficacy score can be calculated. In summary, the survey contains the questions required to assess self-efficacy described by Bandura [16], yet adapted for a rural medical school career assessment [10].

#### Rural clinical school impact on wellbeing

This was measured via a single question (using five point Likert scale) ‘overall my rural clinical school (RCS) placement impacted positively on my wellbeing’. The variable was dichotomized into Strongly disagree/disagree/Neutral (Low) and Strongly agree/agree (high) categories.

#### Supervision

Students’ opinion of their clinical supervisors was assessed using five point Likert scale responses (1 = strongly disagree to 5 = strongly agree) to statements about their supervisors’ behaviours. These questions were based on recognised effective teaching behaviours of rural family medicine preceptors [17]. The sum of these 14 statements was used to produce a variable called ‘total clinical supervisor score’ with a maximum possible score of 70.

#### Other demographic variables

Included gender and whether students had a preexisting rural background on entry to the program (yes/no); Type of location lived longest in Australia (response selection included Capital or major city; Inner regional city or large town (25,000–100,000); Outer regional or smaller town (10,000–24,999); Small remote community (< 10,000); Very remote centre/area).

#### Intrinsic factors

Intrinsic factors were adjusted for in models including whether students chose their RCS location for rural clinical training and whether students were feeling academically supported by rural clinical school during their rural attachment. We also explored perceptions of being financially supported during the rural placement (by the medical school) and a global question on overall support by the rural clinical school they attended.

### Statistical analyses

Statistical analysis was performed using SPSS Version21 (SPSS IBM, New York, USA). Simple logistic regression was used to estimate crude odds ratio to test the association of social isolation with the independent variables. Variables that were significant in the univariate analyses were considered for entry into the multivariate model that predicted intention to practice in rural areas. It was a priori decided to keep gender and rural background were kept in these multivariate models. Supervision ratings and self- efficacy ratings were used in logistic regression models. Independent factors were entered into the model in three stages: 1) social isolation 2) social isolation, gender, rural background, preferred RCS, overall feeling supported by RCS, supervision, impact of RCS on wellbeing 3) rural self-efficacy. *P* values of less than 0.05 were considered as statistically significant.

## Results

The response rate was 81.3% (644/788). In total, 644 medical students completed the survey, 619 were included in this analyses based on availability primary study variables. Table 1 summarises the characteristics of the sample. Although 78.8% of students reported the RCS had positively impacted on their well-being, 31.3% reported feeling socially isolated during their rural placement.

**Table 1:** Characteristics of the sample (*N* = 619)

Characteristics		*N*	%
Gender	Male	265	42.5%
	Female	354	56.7%
Rural background	No	331	53.0%
	Yes	286	45.8%
Type of location living longest in Australia	Capital city	279	44.7%
	Major city	62	9.9%
	Regional	89	14.3%
	Rural	76	12.2%
	Small rural	99	15.9%
	Remote	9	1.4%
Preference for RCS for Clinical training	Last choice	28	4.5%
	Low on list	35	5.6%
	Mid-choice	49	7.9%
	High on list	83	13.3%
	First choice	424	67.9%
Overall RCS Impacted positively on well-being	Strongly disagree/Disagree/Neutral	131	21.2%
	Strongly agree/Agree	486	78.8%
Preferred location for work	Capital/Major city	216	34.6%
	Regional	227	36.4%
	Rural	126	20.2%
	Small rural	36	5.8%
	Remote	13	2.1%

Percentages may not add up to 100% because of missing data

Gender, rural background and feeling financially supported by RCS were not associated with social isolation (Table 2). Factors adjusted for in models and found to be associated with a lower social isolation were: RCS being their first choice for clinical training, feeling overall well-supported by RCS especially academic support, and positive experiences with supervision. Furthermore, students were less likely to feel socially isolated with the presence of higher rural self-efficacy or when students reflected that the RCS impacted positively on their wellbeing.

**Table 2:** Factors associated with social isolation during rural clinical training

	Social Isolation			
		*N* (%)	OR (95% CI)	*p* value
Gender	Male	90 (34.0%)	1.0	
	Female	103 (29.4%)	0.8 (0.6–1.1)	0.25
Rural background	No	105 (31.8)	1.0	
	Yes	86 (30.4)	0.9 (0.6–1.3)	0.72
Type of location living longest in Australia	Capital city/Major city	106 (31.2)	1.0	
	Regional	30 (34.1)	0.8 (0.4–1.4)	0.48
	Rural/Remote	53 (29.0)	1.0 (0.6–1.7)	0.79
Preference for RCS for Clinical training	Others	83 (42.8)	1.0	
	First choice	108 (25.7)	0.5 (0.3–0.6)	< 0.001
RCS Support				
Supported academically by RCS	Strongly disagree/Disagree/Neutral	43 (44.3)	1.0	
	Strongly agree/Agree	151 (28.9)	0.5 (0.3–0.8)	0.004
Supported financially by RCS	Strongly disagree/Disagree/Neutral	79 (35.3)	1.0	
	Strongly agree/Agree	115 (29.0)	0.7 (0.5–1.0)	0.12
Overall well-supported by RCS	Strongly disagree/Disagree/Neutral	48 (46.6)	1.0	
	Strongly agree/Agree	145 (28.1)	0.4 (0.3–0.7)	< 0.001
Rating of Clinical Supervisors	Lower tertile	77 (36.0)	1.0	
	Middle tertile	63 (31.0)	0.8 (0.5–1.2)	0.28
	Upper tertile	50 (26.6)	0.6 (0.4–0.9)	0.04
Rural Self-efficacy	Lower tertile	95 (40.9)	1.0	
	Middle tertile	62 (29.2)	0.5 (0.4–0.8)	0.01
	Upper tertile	33 (20.0)	0.4 (0.2–0.6)	< 0.001
RCS Positively impact on well-being	Strongly disagree/Disagree/Neutral	70 (53.4)	1.0	
	Strongly agree/Agree	124 (25.5)	0.3 (0.2–0.4)	< 0.001

Table 3 displays the multivariate step-wise logistic regression. Model A shows that social isolation led to decreased odds of rural career intentions (OR 0.6 (95% CI 0.4 to 0.8)). Model B demonstrates that social isolation was still associated with reduced rural career intent (OR 0.7 (95% CI 0.4 to 0.9)) after controlling for gender, rural background, RCS placement as a 1st choice, feeling overall supported by RCS, higher supervision ratings and reporting that RCS positively impacted on wellbeing.

**Table 3:** Logistic regression analysis for the effect of self-efficacy on rural career intention

	Intention to practice in rural areas		
	Model A	Model B	Model C
	OR (95% CI)	OR (95% CI)	OR (95% CI)
Social Isolation	0.6 (0.4–0.8)	0.7 (0.4–0.9)	0.6 (0.4–1.1)
Gender (Female)		1.3 (0.9–1.9)	1.3 (0.9–1.9)
Rural background		2.4 (1.6–3.6)	2.0 (1.3–3.0)
Preferred RCS for clinical training		3.0 (1.9–4.9)	2.7 (1.6–4.6)
Overall well-supported by RCS		0.7(0.4–1.4)	0.8 (0.4–1.6)
Supervision		1.3 (1.0–1.7)	1.1 (0.9–1.5)
RCS positively impacted on well-being		0.8 (0.5–1.5)	0.6 (0.3–1.1)
Rural self-efficacy			2.0 (1.6–2.8)

The strongest predictor for rural career intent in Model B (independent of social isolation as the primary outcome) was RCS being 1st choice (OR 3.0 (95% CI 1.9 to 4.9)), followed in descending order by rural background (OR 2.4 (95% CI 1.6 to 3.6)), supervision (OR 1.3 (95% CI 1.0 to 1.07)) and social isolation (OR 0.7 (95% CI 0.4 to 0.9)).

Interestingly, the association between social isolation and rural intent (OR 0.6 (95% CI 0.4 to 1.1)) disappears with the inclusion of rural self-efficacy suggesting rural self-efficacy can be a potential mediating factor (Model C). Furthermore, RCS being 1st choice (OR 2.7 (95% CI 1.6 to 4.6)) and rural background remained significant in the final model (OR 2.0 (95% CI 1.3 to 3.0)).

## Discussion

A primary finding in our studies is that up to 30% of Australian rural clinical students self-report social isolation during their rural clinical placement. We also demonstrated in those students who felt socially isolated were less likely to have rural medical practice intentions. This association was independent of gender, personal wellbeing, rural background, rural clinical school preference, rural clinical support and supervision. However higher levels of self-efficacy could modulate the association of the social isolation on rural career intent.

We note that the specific questions in our survey do not capture all the reasons behind a medical student feeling socially isolated during a rural placement. In our study, we found social isolation was negatively associated with pre-existing intent to study in a RCS (RCS being 1st Choice). Other factors also included the RCS experience, such as feeling supported by RCS or feeling the RCS positively impacted their wellbeing. The decision to choose an RCS placement has been found as a marker of rural career intention, for students of both rural and metropolitan backgrounds [18].

The self-reported social isolation in the context of our study is a subjective experience. Previously it has been suggested that perceived or subjective determinants of social isolation are better predictors of health and behavioral outcomes, when compared to objective measures of social isolation [19, 20]. For example, previous studies have demonstrated that perceived social isolation may predict various outcomes related to quality rather than quantity of social interactions [21]. In the present study we have considered social isolation as a single construct. We have noted in our previous research that other subjective cognitive constructs such as perceived self-rated health is a valuable predictor in measuring patient health seeking behavior [22]. We suggest that a single predictive social cognition marker, can thus be effective without needing to understand underlying mechanisms such as, for example, whether perceived social isolation was due to loneliness.

Interestingly, our study findings suggest that rural self-efficacy can be a potential mediating factor for the relationship between social isolation and rural career intent. Rural self-efficacy is a relatively new construct and has also been shown to be associated with rural career intent [10]. In our study models we demonstrated that social isolation is no longer significantly associated with rural career intent when rural self-efficacy was introduced into the model. We suggest, those reporting to be socially isolated, may be less interested in rural practice and or engage less. Alternatively those socially isolated may have a lower self-efficacy for rural practice or medicine at baseline. Lower self-efficacy (for rural clinical practice) may be associated with increased subjective social isolation. We appreciate that the relationship between rural medical self-efficacy and self-reported social isolation is complex. In particular we note that rural background is also associated with higher rural self-efficacy [10], although as suggested rural background is not associated with perceived social isolation and did not interact with our model outcomes.

### Study limitations and strengths

Causality cannot be determined due to the cross-sectional nature of the study. The broad concept of social isolation reported at the end of a RCS placement is a measure and does not measure underlying causes for the feeling or being more or less socially isolated in the present study. Students may differ in their perceptions of what constitutes wellbeing and social isolation. It is well recognized that subjective measures are difficult to measure directly in cognitive neuroscience. Ideally, a study that included a comparable retrospective time point if they felt more or less socially isolated in the urban area would be beneficial. Strengths of the study included being a national study, and including a wide variety of university rural clinical school programs of varying length, rural background and student maturity.

### Implications

This study may assist universities and rural clinical schools in better understanding the complex relationship between social isolation, rural self-efficacy and future rural career intent among medical students. Our initial findings could also assist policy makers in developing rural workforce strategies that both identify and reduce subjective social isolation for rural medical students. We note that social isolation in one individual can affect other individuals in a group via negative emotions such as loneliness and hence there is benefit to reduce negative outcomes via contagion [23].

## Conclusion

In summary, the self-perception of social isolation during a rural clinical placement by Australian medical students was significant with nearly one-third of all students recalling the perception of being socially isolated. During a rural clinical placement, social isolation has a negative association on student rural practice intentions in our models. Rural self-efficacy may be shown in the future to mediate the association of social isolation in rural medical students on rural clinical workforce intentions.

## Abbreviations

FRAME: Federation of Rural Australian Medical Educators
RCS: Rural Clinical School
